# Image-based recognition of surgical instruments by means of convolutional neural networks

**DOI:** 10.1007/s11548-023-02885-3

**Published:** 2023-05-18

**Authors:** Jan Lehr, Kathrin Kelterborn, Clemens Briese, Marian Schlueter, Ole Kroeger, Joerg Krueger

**Affiliations:** 1 Automation Technology, Fraunhofer IPK, Pascalstr. 8-9, 10587 Berlin, Germany; 2 Central Sterile Services Department, Charité CFM Facility Management GmbH, Augustenburger Platz 1, 13353 Berlin, Germany; 3 Industrial Automation Technology, TU Berlin, Pascalstr. 8-9, 10587 Berlin, Germany

**Keywords:** Object recognition, Surgical instruments, Convolutional neural networks, Instrument tracking

## Abstract

**Purpose:**

This work presents a novel camera-based approach for the visual recognition of surgical instruments. In contrast to the state of the art, the presented approach works without any additional markers. The recognition is the first step for the implementation of tracking and tracing of instruments wherever they are visible and could be seen by camera systems. Recognition takes place at item number level. Surgical instruments that share the same article number also share the same functions. A distinction at this level of detail is sufficient for most clinical applications.

**Methods:**

In this work, an image-based data set with over 6500 images is generated from 156 different surgical instruments. Forty-two images were acquired from each surgical instrument. The largest part is used to train convolutional neural networks (CNNs). The CNN is used as a classifier, where each class corresponds to an article number of the surgical instruments used. Only one surgical instrument exists per article number in the data set.

**Results:**

With a suitable amount of validation and test data, different CNN approaches are evaluated. The results show a recognition accuracy of up to 99.9% for the test data. To achieve these accuracies, an EfficientNet-B7 was used. It was also pre-trained on the ImageNet data set and then fine-tuned on the given data. This means that no weights were frozen during the training, but all layers were trained.

**Conclusion:**

With recognition accuracies of up to 99.9% on a highly meaningful test data set, recognition of surgical instruments is suitable for many track and trace applications in the hospital. But the system has limitations: A homogeneous background and controlled lighting conditions are required. The detection of multiple instruments in one image in front of various backgrounds is part of future work.

## Introduction

**Fig. 1:**
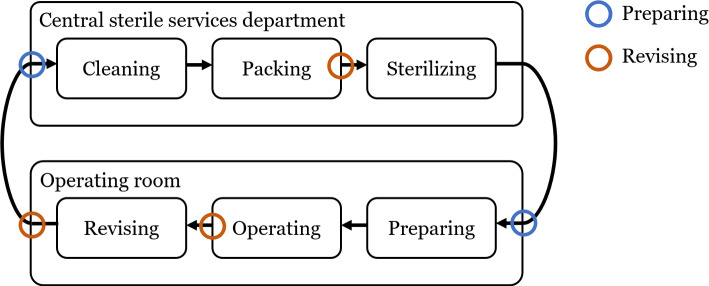
Cycle of surgical instruments within a hospital

The tracking and recognition of surgical instruments plays a crucial role in many clinical and medical processes. Knowing which instrument was at a particular point of time during an operation can contribute to the success of the operation and provide clarity in case of failure. Furthermore, this information also serves the direct preparation and revising of the surgery in the operating room itself. They also help to improve the efficiency of the processes in a central sterile services department (CSSD). Figure 1 shows a typical cycle of reusable surgical instruments in hospitals.

Surgical instruments are transported in so-called trays between operating rooms and the CSSD. It is precisely defined which instruments belong to which tray. The lack of a surgical instrument or the presence of a wrong instrument can risk the success of an operation and must be avoided in all preparation processes [1]. In follow-up processes, the missing of a surgical instrument immediately after the operation can have serious consequences. If the instruments are mixed in the trays after an operation, the sterilization processes significantly lose efficiency. In consequence, the recognition and tracking of surgical instruments is an important aspect that can be prone to mistakes if carried out purely by humans.

**Fig. 2:**
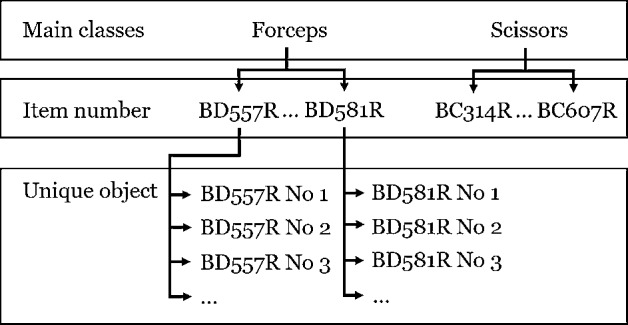
Classification of surgical instruments with respect to different levels of detail

It is important to distinguish the level of detail in which the recognition of surgical instruments takes place. A simple breakdown of instruments into main classes provides no added value. Whether the instrument is a pair of scissors or a clamp is basically an important information. But not all clamps fulfill the same function. An assignment of the manufacturer and the item number is necessary to ensure the required functions of the instruments for an operation. In contrast, a unique identification is not necessary, since instruments that have the same item number are basically the same medical device and therefore provide the same function (cf. Figure 2). In this work, it is first investigated whether the partially very similar but also very different surgical instruments can be recognized under controlled environmental conditions.

### Scientific question

In cooperation with Charité CFM Facility Management GmbH, the following scientific question was investigated in this work: Is image-based recognition of surgical instruments at item number level possible using only machine learning and image processing methods?

## State of the art

The state of the art addresses current techniques and systems that are used marker-based recognition, track and trace of surgical instruments. The field of object recognition with image processing methods based on machine learning is also reviewed, especially convolutional neural networks (CNNs).

The authors are not aware of any publication or method that has successfully tested surgical instrument recognition based on convolutional neural networks to date.

### Marker-based technologies

Marker-based recognition means that an additional element must be added to each instrument so that a technical system can recognize this instrument. A separation is made between non-image processing technologies and image processing technologies.

#### Non-image processing technologies

Non-image processing technologies use radio frequency identification (RFID) techniques. These systems consist of a reader and a passive transponder for each instrument. The reader can contactlessly read the passive transponder and thus identify and localize the transponder. There are already established products available on the market for this purpose [2–6].

#### Image processing technologies

Image processing technologies use visual markers as QR codes or bar codes [7]. Several publications address the track and trace of surgical instruments with image processing. But the focus is on the use in the operating room during an operation. Therefore, the motion of an object is tracked. The knowledge about the item number of an instrument must be set manually in a previous step [9–11].

#### Advantages and disadvantages

The technologies presented have the advantage that they can identify each instrument uniquely, i.e., much more precisely than the item number-wise recognition. The disadvantage is that such detailed recognition is often not necessary and each surgical instrument must be specially prepared for this purpose. Due to the sterilizing process of the instruments, markers such item numbers, QR codes or bar codes wear out and are no longer readable. In addition, modified instruments must be re-certified in order to be used.

### Marker-free technologies

Numerous publications have already shown that modern methods of image processing from the field of machine learning achieve great success in object recognition [8, 12–15]. The methods recognize objects simply based on their visual appearance. Recognition is formulated as a classification problem. An image is assigned to a class, i.e., an item number.

So-called convolutional neural networks (CNNs) perform feature extraction and subsequent classification using a holistic approach. Their special advantage is that no manual modification of weights or parameters is necessary. The CNN learns all weights during a training process using a large amount of training data. The network architectures used for this work are presented below.

#### Density networks

The density network (short: DenseNet) is characterized by the fact that the features of all preceding layers are available as input for each layer [13]. This results in a very high density of information. Classically, CNNs only receive the features of the previous layer in each layer. This has the disadvantage that calculations in CNNs take place several times and the same features can be calculated several times at different locations in the network. The DenseNet can thus calculate better features more efficiently. The authors compare different networks sizes with respect to the number of layers (the depth of the network). The version with 201 layers gives the best performance between recognition accuracies and size of the network. Therefore, DenseNet-201 is chosen for this work.

#### Efficient networks

The efficient network (short: EfficientNet) is a partially automated CNN architecture [14]. Machine learning algorithms have optimized an already existing architecture at various points: width, depth and resolution of the network. The authors present eight different variants with respect to the number of weights of the network. The network with the highest number of weights is version B7. It reaches the highest recognition accuracies. Therefore, EfficientNet-B7 is chosen for this work.

#### Big transfer models

The big transfer model (short: BiT-M) is a modified version of a residual network [15]. Its special feature is that it was pre-trained on a data set comprising 21 million images [13].

## Concept

The presented work addresses the recognition of surgical instruments on images. The use of machine learning methods requires the acquisition of training, validation and test data. This section describes how these data are obtained and provided. Furthermore, the design of experiments is presented.

### Acquisition setup

An acquisition system with three industrial cameras is used for data acquisition. The cameras focus on the instrument at three different angles: Top view = $0^\circ$, 2nd view = $30^\circ$ and 3rd view = $45^\circ$. A $90^\circ$ view for a side view was omitted, since the instruments are characterized mainly in length and width which is addressed with the top view. Essential recognition features are not expressed in height for most of the instruments. The instrument tray is homogeneous, white and non-reflective. The lighting is diffused to avoid shadows cast by the instruments. The colored RGB images have a resolution of $896 \times 1024$ pixels (width $\times$ height).

### Data set

**Fig. 3:**
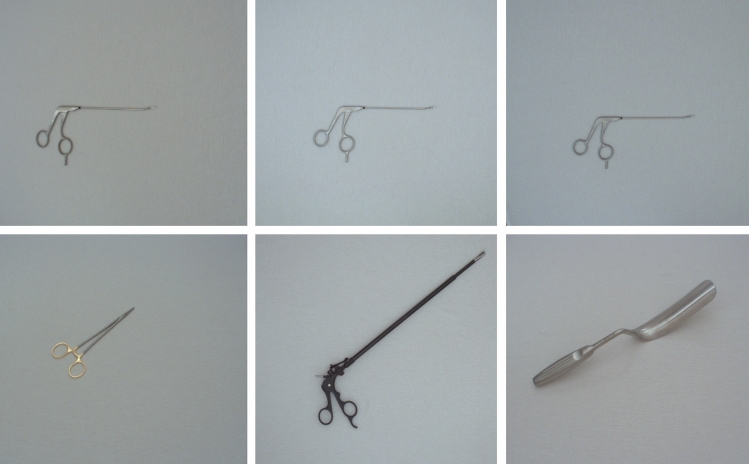
The top row shows surgical instruments with different article numbers that are very similar. The bottom row shows surgical instruments that look very different

**Fig. 4:**
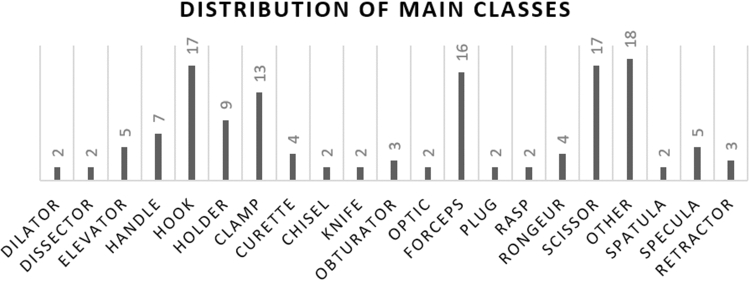
Distribution of the main classes for the used instruments in this work

A total of 156 different surgical instruments are used in this work. They provide a wide range of sizes and types. Figure 3 shows example images of surgical instruments which look very similar and very different. Fig. 4 shows the distribution of main classes across all used instruments.

**Fig. 5:**
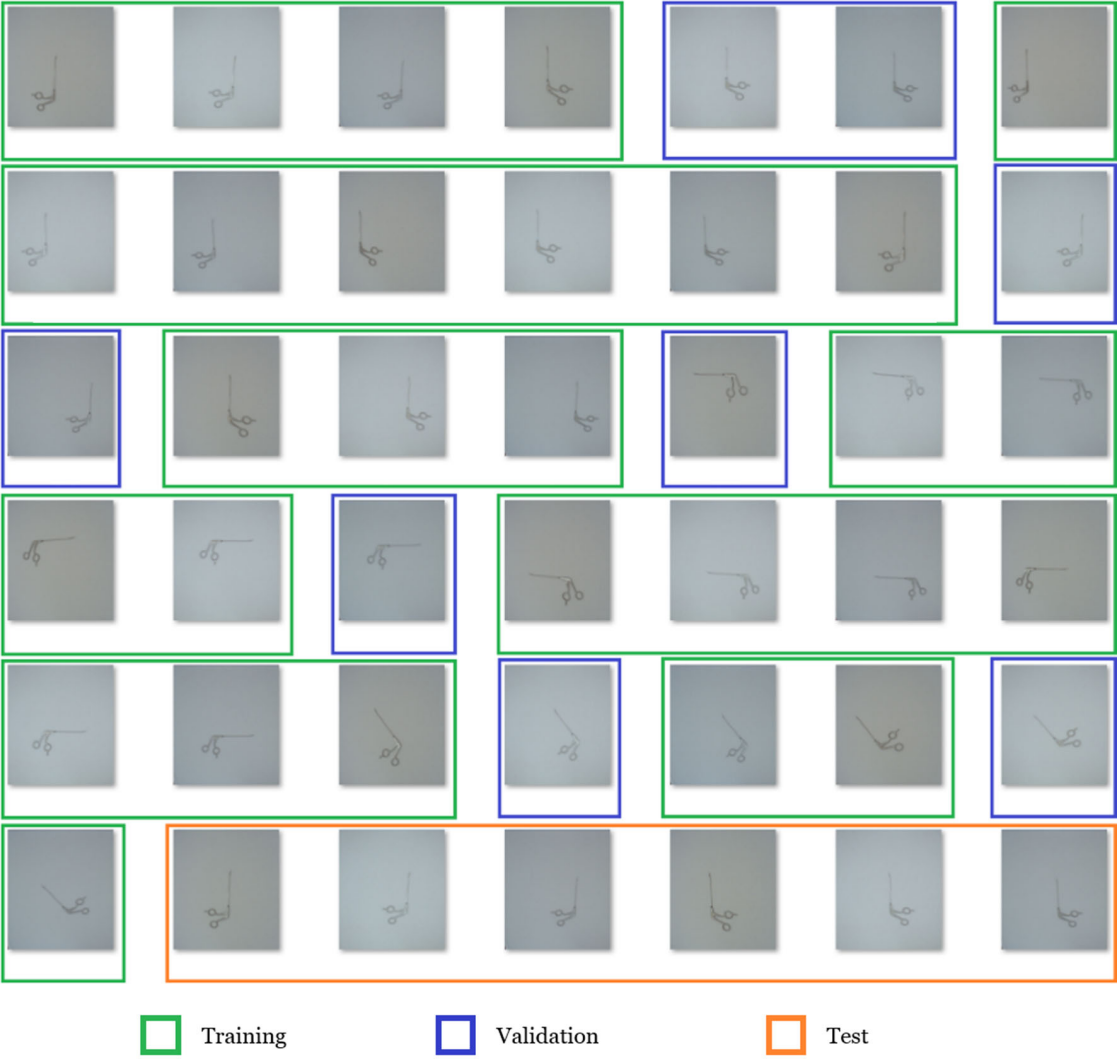
The 42 images for one single instrument are shown here. The colored boxes show which images are used for training, validation and test data. Details can be found in the following section

A total of seven different positions and two different orientations were used to cover all perspectives of the instruments. Using three cameras, this results in $7 \times 2 \times 3 = 42$ images per instrument. Only one instrument is presented to the acquisition system at any given time. The instrument is captured with different orientations (e.g., lying on its side) and in different positions (e.g., central or left in the image). The entire image set of one instrument is shown in Figure 5. Note that the first and seventh positions are very similar as they describe the intuitive position.

To train the CNNs, the data set is split as follows:

1. *Training data* These data are used to learn the patterns of objects to be recognized. The CNNs use the training data to automatically tune their weights during the training phase.
2. *Validation data* About 25% of the training data is randomly selected and held out to the CNN during training. This allows testing how the CNN behaves with unknown data. The validation data does not follow a fixed scheme. The higher the recognition accuracy in the validation data the higher the generalization of the algorithm.
3. *Test data* These data consist of six images per instrument and describe the intuitive position of how a worker would present an instrument to the acquisition system (straight orientation, central in the image). The higher the recognition accuracy in the test data, the higher the specialization of the algorithm to the use case.

The total amount of 6552 images is split into 4496 training images, 1120 validation images and 936 test images (percentage distribution: 67/17/14). The final recognition accuracy for the test data is the deciding criterion.

### Design of experiments

The CNNs presented in “Marker-free technologies” section consist of several million network weights, all of which are tuned automatically in the training process. In addition to architecture selection and training duration, different optimization techniques will also be evaluated.

#### Prior knowledge

This is the targeted input of prior knowledge to optimize feature extraction even with little data. The CNNs are pre-trained on the publicly available ImageNet data set [16]. Three different ways are distinguished in the experiments:

1. *Random initialization* The CNN is initialized with random weights and parameters.
2. *Pre-trained* The CNN is pre-trained on the 1.2 million images of the ImageNet data set. The feature extraction is frozen. Only the classifier is trained.
3. *Fine-tuning* The initialization is done as in the “pre-trained” case. The feature extraction is not frozen but adapted to the instruments with fine-tuning during the training process.

#### Data augmentation

All training images are modified using image processing techniques. During training, the images are randomly rotated or their color values (e.g., hue, saturation, contrast) are changed by up to 5%.

## Experiments

The baseline for all evaluations is defined by DenseNet-201. The training is initially trained over 50 epochs, so the training data is presented to the CNN 50 times. The corresponding accuracies for the test data are entered in Table 1. First, the extent to which the use of prior knowledge provides an advantage in the recognition of surgical instruments is evaluated.

It is obvious that the resulting data set is far too small to train a network from scratch. Using prior knowledge helps a lot, but still gives worse results. Only with the fine-tuning approach, very good results are achieved. In the test data, even a Top 5 accuracy of 100% is achieved. This means that correct item number is within the first five predictions of the CNN. The fine-tuning approach is therefore always used for the following calculations.

To ensure the robustness of the recognition even with increased variance, it is advisable to augment the training data set. In each epoch of the training, a uniform distribution is used to randomly project these augmentations onto each image. Thus, the variance within the training data increases significantly. Table 2 shows that expanding the training data in terms of rotation is effective. In contrast, increasing the variance by slightly manipulating the pixel values does not add any value.

**Table 1:** Evaluation for the usage of prior knowledge

Method	Validation		Test	
	Top 1 (%)	Top 5 (%)	Top 1 (%)	Top 5 (%)
Random initialization	4.6	12.3	4.7	14.0
Pre-training	45.6	76.9	58.0	86.1
Fine-tuning	**91**.**8**	**100**.**0**	**95**.**3**	**100**.**0**

**Table 2:** Evaluation of data augmentation techniques

Method	Validation		Test	
	Top 1 (%)	Top 5 (%)	Top 1 (%)	Top 5 (%)
None	91.8	**100**.**0**	95.3	**100**.**0**
Rotation	**97**.**8**	**100**.**0**	**98**.**9**	**100**.**0**
Color jitter	94.1	99.9	98.3	**100**.**0**

Table 3 shows the accuracies of a DenseNet-201 for different training lengths. It can be seen that the difference between 20 and 50 epochs is hardly relevant. But a training over 500 epochs brings a significant increase in recognition.

**Table 3:** Evaluation of the duration of the training

Epochs (duration [h])	Validation		Test	
	Top 1 (%)	Top 5 (%)	Top 1 (%)	Top 5 (%)
20	91.5	99.6	95.3	99.8
50	91.8	**100**.**0**	95.3	**100**.**0**
100	94.3	**100**.**0**	97.9	**100**.**0**
200	94.8	**100**.**0**	97.9	**100**.**0**
500	**95**.**9**	**100**.**0**	**98**.**5**	**100**.**0**

Finally, different architectures are evaluated against each other. All methods are trained without data augmentation over 50 epochs. The BiT-M again illustrates the influence of transfer learning. Table 4 shows the increase compared to the DenseNet-201 with 5.7% points in the Top 1 accuracy for the validation data and 4.1% points for the test data. EffiecientNet-B7 achieves the highest values for the Top 1 accuracy of the validation data and the test data in this work. The Top 1 accuracy of the test data with 99.9% means in absolute numbers that there is only one misclassification in the 936 test images.

**Table 4:** Evaluation of different network architectures

Epochs (duration [h])	Validation		Test	
	Top 1 (%)	Top 5 (%)	Top 1 (%)	Top 5 (%)
DenseNet-201	91.8	**100**.**0**	95.3	**100**.**0**
BiT-M	97.5	**100**.**0**	99.4	**100**.**0**
EfficientNet-B7	**98**.**5**	**100**.**0**	**99**.**9**	**100**.**0**

## Discussion

It is obviously that the specific integration of prior knowledge is necessary in order to be able to use CNNs in the scenario described. This is mainly due to the size of the networks. To train all the weights with 4496 training images is not possible. Initializing the CNNs with meaningful weights so that only fine-tuning has to be done, is very successful. Another method to address the small amount of training data is to augment the data artificially. For example, the images can be rotated or their pixel values can be changed. Enhancing the training data with rotated images has led to an increase in recognition accuracy. Recognition of instruments is a use case that requires high specialization rather than high generalization of CNNs. The longer an algorithm trains the higher the recognition accuracy. The highest increase is found in the first 10 epochs. Nevertheless, it has been shown that longer training times can still achieve small but measurable increases in recognition accuracy. For future work, it must be noted that the training duration per epoch increases proportionally to the number of training dates.

## Conclusion

A total of 156 different surgical instruments are captured and a data set with a total of 6552 images is created. The instruments can be recognized with a maximum Top 1 accuracy of 99.9% and a Top 5 accuracy of 100%. The scientific question of “Scientific question” section can be answered with yes.

This work is the basis for an image-based tracking of surgical instruments in the operating room during an operation. It addresses the recognition of one instrument per image in front of a homogeneous background with controlled lighting conditions. The high variance of possible backgrounds and scenes as well as the very small ratio of instrument size to image size pose the most difficult challenges. Overlapping instruments or covering by operating room personnel are further hurdles to reliable track and trace of instruments in the operating room. It should be noted that the established marker-based technology can only be replaced where recognition at item number level makes sense and where instruments are visible.
